# Estrogen, Angiogenesis, Immunity and Cell Metabolism: Solving the Puzzle

**DOI:** 10.3390/ijms19030859

**Published:** 2018-03-15

**Authors:** Annalisa Trenti, Serena Tedesco, Carlotta Boscaro, Lucia Trevisi, Chiara Bolego, Andrea Cignarella

**Affiliations:** 1 Department of Pharmaceutical and Pharmacological Sciences, University of Padua, 35131 Padua, Italy; annalisa.trenti@gmail.com (A.T.); carlotta.boscaro@phd.unipd.it (C.B.); lucia.trevisi@unipd.it (L.T.); chiara.bolego@unipd.it (C.B.); 2 Venetian Institute of Molecular Medicine, 35129 Padua, Italy; serena.tedesco1988@gmail.com; 3 Department of Medicine, University of Padua, 35128 Padua, Italy

**Keywords:** estrogen, 17β-estradiol, angiogenesis, metabolism, endothelium, macrophages, immune response

## Abstract

Estrogen plays an important role in the regulation of cardiovascular physiology and the immune system by inducing direct effects on multiple cell types including immune and vascular cells. Sex steroid hormones are implicated in cardiovascular protection, including endothelial healing in case of arterial injury and collateral vessel formation in ischemic tissue. Estrogen can exert potent modulation effects at all levels of the innate and adaptive immune systems. Their action is mediated by interaction with classical estrogen receptors (ERs), ERα and ERβ, as well as the more recently identified G-protein coupled receptor 30/G-protein estrogen receptor 1 (GPER1), via both genomic and non-genomic mechanisms. Emerging data from the literature suggest that estrogen deficiency in menopause is associated with an increased potential for an unresolved inflammatory status. In this review, we provide an overview through the puzzle pieces of how 17β-estradiol can influence the cardiovascular and immune systems.

## 1. Setting the Stage: Estrogen, the Cardiovascular System and the Immune Response

In addition to its essential role in sexual development and reproduction in females, estrogen is involved in a wide range of physiological processes in different tissues [1], even in male subjects. Evidence accumulated over the years demonstrated that estrogen has protective effects on the cardiovascular system [2,3,4], mainly related to interaction with multiple cell types including immune cells, such as B lymphocytes and macrophages [5] and vessel wall cells, including smooth muscle [6,7] and endothelial cells [8,9,10,11].

In women, estrogen circulating levels fluctuate during the menstrual cycle and its concentration changes in relation to age [12]. The most important estrogen circulating from menarche to menopause is 17β-estradiol (E2). Close to menopause, estrogen plasma levels decrease compared to those present in fertile women [13] and become equivalent to those present in men. However, E2 continues to be synthesized, starting from androgens, in extragonadal sites such as breast, brain, muscle, bone and adipose tissue where it acts locally as a paracrine or autocrine factor [14]. Declining estrogen levels are associated with a variety of metabolic changes and cardiovascular diseases [15]. The metabolic effects mediated by estrogen take place in multiple tissues including skeletal muscle and liver [16].

E2 prevents endothelial dysfunction, vascular inflammation and atherosclerosis [15]. In addition, available evidence points to E2 as a key factor in promoting endothelial healing and angiogenesis [8,9,10,17] through endothelial progenitor cells, immune inflammatory cells and platelet mobilization, which contribute synergistically to endothelial repair [18,19,20]. The important role of E2 in the angiogenic process is also noticed in ischemia-reperfusion tissue injury, where E2 induces the formation of collateral vessels [21]. Angiogenesis stimulation by E2 accelerates functional endothelial recovery after arterial injury, which could be beneficial in coronary artery disease, peripheral arterial disease, cerebral ischemia and congestive heart failure [21]. The direct actions of E2 on endothelial cells contribute to accelerate re-endothelialization in vivo [22]. This process following endothelial damage [20,22] is accompanied by reduced neointima formation as a result of inhibition of smooth muscle cell proliferation and migration [23]. Furthermore, E2 promotes the natural resolution of inflammation in wound healing [24].

The immune system demonstrates remarkable sex differences: females tend to have a more responsive immune system compared to their male counterparts. The outcome and survival rates from e.g., infections or sepsis are sometimes better in females than in males [25]. Females, however, respond more aggressively to self-antigens and are more susceptible to autoimmune diseases [26]. The body of human data on gender differences in immune response is rapidly growing. Amadori and colleagues [27] were the first to demonstrate that circulating T lymphocytes in fertile women are more abundant than those in men; this also occurs in other female mammals, suggesting a common trait in different species that endows females with a more rapid and efficient immune response [28,29]. It has long been recognized that steroid hormones play a role in the regulation of the immune response to infection or tissue damage and modulate all levels of the innate (neutrophils, macrophages/monocytes, natural killer cells, dendritic cells) and adaptive immune systems (T and B cells) (reviewed in [30]). Estrogen has been shown to regulate neutrophil number and function, and the production of chemokines such as monocyte chemoattractant protein (MCP)-1 and cytokines including tumor necrosis factor (TNF)-α, interleukin (IL)-6 and IL-1β. On the other hand, since ovarian activity decreases and eventually stops with aging, several disease conditions may show up, which are characterized by a strong inflammatory component associated with the post-menopausal state [26,31].

The aim of this review is to discuss the multifaceted role of estrogen in vascular biology and in the immune response, particularly in the monocyte/macrophage system, and to further integrate available evidence (i.e., solve the puzzle) regarding the estrogenic control of double-edged processes such as angiogenesis and metabolism.

## 2. Estrogen Receptors

The effects induced by estrogen in different tissues are the result of the activation of transcriptional and non-transcriptional signal pathways. Estrogen exerts both rapid and long-term actions through their binding with ERs [1]. Several ER subtypes have been identified: the nuclear isoforms, ERα and ERβ, and the transmembrane G-protein-coupled receptor 30/G-protein estrogen receptor 1 (GPER1). ERα and ERβ act as transcription factors responsible for many genomic effects, modulating gene expression by direct binding to DNA at specific estrogen response elements (EREs) [32]. In contrast, GPER1 is mainly involved in mediating rapid intracellular responses induced by estrogen [33,34].

The genes encoding ERα, ERβ and GPER1 are *ESR1*, *ESR2* and *GPER1*, respectively. The two intracellular receptors have different molecular weights; in particular, ERα consists of 595 and ERβ of 530 amino acids, respectively [35]. Their structure consists of two main domains: the carboxy-terminal domain for interaction with the ligand (ligand-binding domain, LBD), which contains the activator factor-2 (AF-2), mediating a wide range of functional responses, and the central DNA-binding domain, responsible for binding to EREs [36]. Other regions are involved in transcriptional activation: the transcriptional regulatory domain (constitutively active amino-terminal domain, AF-1) and a hinge domain between the DNA-binding domain and LBD, which gives flexibility to the protein [1,37].

ERα is a ligand-dependent transcription factor that exerts its genomic, also called nuclear actions through binding to chromatin and mobilization of cofactors to influence the transcription of its target genes. A fraction of ERα can elicit membrane signaling (non-genomic effects) by association with the plasma membrane [38,39]. Rapid changes in adenylate cyclase, mitogen-activated protein kinases (MAPK) and phosphatidylinositol 3 kinase (PI3K) activities or in cytoplasmic calcium concentration and endothelial nitric oxide synthase (eNOS) activation constitute established non-genomic effects. By using mice expressing ERα proteins with inactivated genomic or nongenomic signaling, it has been shown that the preserved arterial actions of E2 were membrane-dependent [40], whilst the estrogenic responses of uteri were highly dependent upon the genomic actions of ERα [41]. These studies thus demonstrated for the first time that the respective contributions of nuclear/genomic and membrane effects towards the estrogenic response are tissue-specific. Accordingly, we showed that administration of a selective ERα agonist confers cardiovascular protection dissected from unwanted uterotrophic effects [3], suggesting that ERα-selective agonists represent a potential safer alternative to natural hormones.

The estrogenic membrane receptor GPER1 belongs to the family of G protein-coupled receptors and is characterized by the presence of seven transmembrane helices. The organization of the seven helices involves the amino-terminal portion located outside the cell and the carboxy-terminal portion in the cytoplasm. Cytoplasmic loops are involved in the selective binding and activation of various heterotrimeric proteins [1]. This receptor is expressed at the endoplasmic reticulum and in the plasma membrane [33,42]. E2 binds to GPER1 with nanomolar affinity, in the range of 3–6 nM [33], while its affinity for nuclear receptors is ten times higher, in the range of 0.1–0.4 nM [1]. However, in cells expressing both ERα and GPER1, coordinated signaling is likely to occur, with some evidence supporting this in monocytes [43], ovarian cancer cells [44], uterine stromal cells [45] and coronary vessels [46]. Accordingly, the emerging notion that GPER acts as an autonomous ER in vivo and also interacts with intracellular ERs has been recently reviewed by Romano and Gorelik [47].

## 3. Estrogen Receptors and Endothelial Function

ERα is expressed in the vascular tissue [48,49]; although ERβ distribution in vascular tissues is less characterized, human endothelial cells do express ERβ [50]. ERα has been long recognized to mediate most beneficial cardiovascular effects of E2 [2,3,4], but it is also involved in pathologic cell proliferation in the setting of cancer [51].

Several of estrogen cardiovascular actions are actually mediated by direct effects on the vessel wall resulting in the control of endothelial function and plasma lipid profile. In particular, estrogen increases the synthesis and release of nitric oxide (NO) and prostacyclin, well-known endothelial-derived vasodilators and anti-platelet agents, and negatively regulates production of several pro-inflammatory mediators in situations of vascular injury [52]. More specifically, estrogen upregulates the expression of enzymes involved in prostacyclin biosynthesis, i.e., cyclooxygenase (COX)-1 and prostacyclin synthase, thereby increasing systemic prostacyclin levels in rodents [53]. Moreover, estrogen increases both COX2 expression and prostacyclin generation in ovariectomized low-density lipoprotein receptor null (LDLR−/−) mice and substantially reduces atherosclerotic lesion size [54]. Accordingly, the protective effects of estrogen were abrogated by disruption of the prostacyclin receptor (IP) gene in the double LDLR−/−/IP−/− null mouse, suggesting that the protective actions of estrogen within the cardiovascular system are, at least in part, mediated by endothelial prostacyclin and its receptor, the IP. Notably, a physiological concentration of E2 induces transcription but not translation of COX-2 in human endothelial cells exposed to laminar shear stress [4]. E2 increases NO levels in cerebral and peripheral endothelial cells in vitro via eNOS activation and ER-mediated mechanisms [55]. NO is essential for vascular endothelial growth factor (VEGF)-induced angiogenesis in vitro [56] and in vivo [57]. Recent studies have shown that changes in the relative expression of ERβ/ERα may influence some E2 effects, such as the modulation of vascular NO bioavailability in aging rodents [58].

Endothelial cells also express GPER1 [59], which mediates nongenomic rapid effects including calcium influx, cAMP synthesis or kinase (such as PI3K) activation. These events are involved in the regulation of vascular tone [33,34,60]. Interestingly, a novel role for GPER has emerged in regulating the expression of NADPH oxidase 1 (NOX1), which is essential for reactive oxygen species generation in the cardiovascular system [61]. 

In conclusion, estrogen mediates both rapid and longer-term effects on the vessel wall. Novel vascular target genes regulated by ER subtypes are being identified, thereby providing potential opportunities for pharmacological intervention.

## 4. Estrogen, Angiogenesis and Metabolism

Additional puzzle pieces that need to fit together include estrogen, angiogenesis and metabolism. Migration and proliferation of endothelial cells are closely involved in re-endothelialization and angiogenesis. Angiogenesis consists of a number of subsequent biological events and is a tightly regulated process. In adult organisms, angiogenesis is virtually absent under normal conditions, except in the female reproductive tract, where it is routinely observed in the uterus in association with E2 fluctuations [62]. E2 stimulates endothelial cell proliferation in vitro [8] and in vivo [8,9,17], and inhibits spontaneous, as well as TNF-α-induced, apoptosis [63,64]. Furthermore, E2 enhances adhesion of HUVECs to various matrix proteins and increases cell migration, thus promoting angiogenesis [8,10]. The mechanisms responsible for the proangiogenic effect of E2 have been widely investigated and appear to be largely mediated by ERα activation [65]; accordingly, angiogenesis is impaired in ERα knockout mice [66]. In HUVECs, E2 has been shown to enhance cyclins A and B1 gene expression through involvement of the classical ER pathway [67]. E2 treatment also promotes proliferation and increases RhoA gene expression and activity in an ERα-dependent manner [10,68]. Through a rapid, non-genomic pathway ligand activated by ERα, E2 promotes rearrangements of actin cytoskeleton that allow the formation of specialized cell membrane structures, such as focal adhesion complexes, pseudopodia and membrane ruffles [50]. Estrogen also stimulates VEGF production in uterine and vascular tissue [69,70]. The rapid re-endothelialization induced by estrogen after vascular injury may be due, in part, to increased local expression of VEGF [9,17]. E2-induced increases in VEGF receptor-2 expression on human myometrial microvascular endothelial cells appears to be mediated primarily by ERα [71]. In addition, E2 promotes increased β1, α5 and α6 integrin expression on endothelial cell surface [72] and induces phosphorylation of focal adhesion kinase (FAK) followed by its translocation toward membrane sites, where focal adhesion complexes are assembled [65].

In pathological circumstances, such as breast cancer, a clear association has been made between estrogen, ER expression by endothelial cells, angiogenic activity and/or tumor invasiveness [73]. In this context, transient E2 induction of VEGF results from E2-induced upregulation of the oncogenic nuclear transcription factor c-Myc via ERα activation, whereas estrogen withdrawal in tumors induces hypoxic conditions responsible for VEGF upregulation [74]. Because the expression of glucose transporter 1 (GLUT1) is regulated by c-myc [75], it is conceivable that estrogen interaction with ERα activates c-myc, which in turn up-regulates GLUT-1 expression, thereby affecting tumor perfusion and glucose transport and metabolism through glycolysis. However, recent in vitro and in vivo observations indicate that membrane ERα signaling effects could mediate, or at least potentiate, the beneficial actions of estrogen on energy balance, insulin sensitivity, and glucose metabolism. Indeed, selective activation of the extranuclear ERα pool appears to induce endothelial actions and limit adipose tissue and fatty liver accumulation [76]. Moreover, preliminary data obtained from a mouse model with membrane-specific loss of function of ERα support a significant role of membrane ERα pool and membrane-derived signaling effects in the metabolic protective effects of estrogen [77].

During angiogenesis, endothelial cells must increase their metabolic activity to generate energy quickly and to facilitate the incorporation of nutrients into biomass. De Bock and colleagues [78] demonstrated that phosphofructokinase-2/fructose-2,6-bisphosphatase-3 (PFKFB3)-driven glycolysis regulates vessel branching. PFKFB3 is a direct target of E2 action; in ER-responsive breast cancer cells (MCF-7), E2 promotes PFKFB3 mRNA transcription and up-regulates PFKFB3 protein expression through ERα via direct binding to PFKFB3 promoter [79]. Recently, we demonstrated that the increased angiogenic response in E2-stimulated HUVEC is mediated by enhanced PFKFB3 expression peaking after 3 h, consistent with the activation of a membrane receptor considering that a nuclear/genomic effect would require a longer time. Treatment with the selective GPER1 agonist G-1 mimics the chemotactic and proangiogenic effect of E2 and also increases PFKFB3 expression, suggesting that E2-induced angiogenesis is mediated, at least in part, by the membrane receptor GPER1 [11]. Hence, even if steroid hormones have been classically described to mediate biological effects via intracellular receptors, non-genomic mechanisms of activation through membrane receptors responsible for endothelial cell motility, proliferation, and angiogenesis have also been demonstrated. Additional mechanisms for GPER1-mediated angiogenic stimulation may include the up-regulation of acid ceramidase expression, the increase of X-linked inhibitor of apoptosis protein (XIAP) and the regulation of Na^+^/H^+^ exchanger-1 (NHE-1) activity as reviewed recently by De Francesco et al. [80].

**Figure 1:**
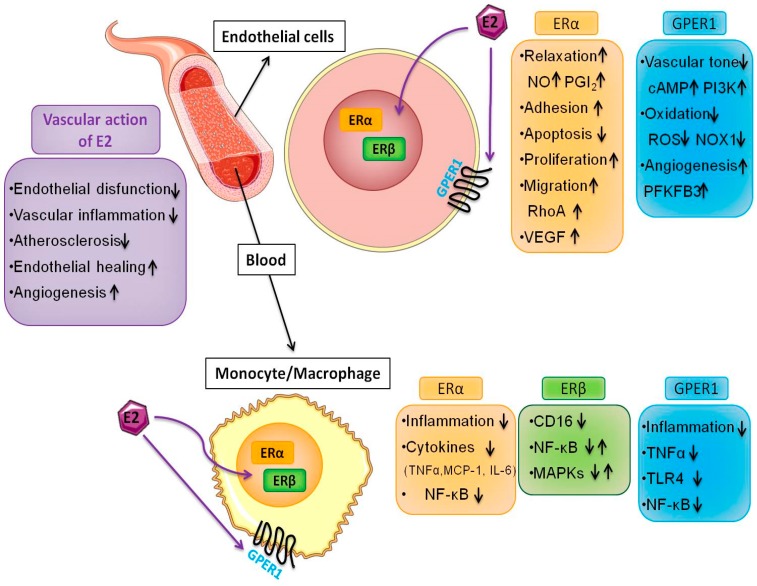
Multiple effects of 17β-estradiol (E2) in endothelial cells and macrophages. E2 induces protective effects on the cardiovascular system by promoting endothelial healing and angiogenesis through various pathways including the acceleration of re-endothelialization in vivo, the induction of proliferation and rearrangements of the actin cytoskeleton. E2 regulates the induction of chemokines and cytokines, and modulates macrophage immune phenotypes. These events are mediated by intracellular and membrane ER subtypes that are operatively linked in several cell types. The interaction between endothelial cells and macrophages is relevant in multiple disease settings such as atherosclerosis and cancer.

Experimental evidence accumulated over the past decade indicates that the direct effect of E2 on endothelial cells explains some cardiovascular benefits of the ovarian sex steroid hormone (Figure 1), but the specific pathways they influence remain to be elucidated. We have unraveled a previously unrecognized mechanism of estrogen-dependent endocrine-metabolic crosstalk in HUVECs which may have implications in angiogenesis occurring in ischemic or hypoxic tissues [11]. However, fitting these puzzle pieces together would require dissecting the molecular mechanisms of estrogen’s proangiogenic effect in different disease contexts such as cancer. Thus, tissue-specific pharmacological control of endocrine-metabolic crosstalk appears to be a rewarding therapeutic strategy.

## 5. Estrogen and Macrophage Function

New data are redefining macrophages as diverse, polyfunctional and plastic cells that respond to the needs of the tissue at steady state and during disturbed homeostasis. Inflammation plays a critical role in the onset and progression of degenerative diseases, and is characterized by activation of tissue-resident macrophages as well as monocyte-derived macrophages that originate and renew from adult bone marrow. Under normal conditions, these cells provide immune surveillance and host defense in tissues to maintain homeostasis. However, upon sensing changes in the microenvironment, macrophages become activated, undergoing a morphological and functional switch [81]. Activation of these cells is not an “all-or-none” process, but rather a continuum characterized by a wide spectrum of molecular and functional phenotypes ranging from the “classical” M1 activated phenotype, with a highly pro-inflammatory profile, to the “alternative” M2 phenotype, associated with a beneficial, less inflammatory, protective profile [82,83]. Accordingly, these new models of activation and classification account for the functional diversity of macrophages that is relevant in vivo both in health and disease conditions including obesity, autoimmunity and neurodegeneration [84]. For instance, since a prominent feature of tissue remodeling is neoangiogenesis, macrophage polarization could affect the angiogenic process [85,86], which in turn is a determinant of adipose tissue expansion during obesity [87].

Estrogen has been shown to act as regulator of the immune function of the monocyte-macrophage system, especially regarding the production of cytokines. For instance, estrogen treatment in ovariectomized animals reduces expression of vascular MCP-1 and leukocyte infiltration into injured tissues, such as arteries and lung [88,89]. Estrogen affects the activation of nuclear factor kappa-light-chain-enhancer of activated B cells (NF-κB) in monocytes derived from umbilical cord blood, suggesting that high E2 concentrations during gestation affect the immune response in newborns [90]. Later in life, the production of cytokines by monocyte/macrophages is heavily influenced by the ovarian cycle, oral contraceptive use and estrogen replacement [91,92]. In vitro pre-treatment with E2 of human macrophages inhibits the NF-κB signaling pathway and the production of TNF-α induced by lipopolysaccharide (LPS) [93]. Estrogen has been also shown to enhance production [94] and prevent degradation of the endogenous NF-κB inhibitor IκB-α [95]. Other authors have reported the inhibitory effect of E2 on the production of pro-inflammatory cytokines [96]. By contrast, chronic exposure of murine macrophages to E2 in vivo increases production of pro-inflammatory cytokines (e.g., IL-1β, IL-6, TNF-α) [97,98].

Macrophages have long been recognized as crucial regulators of vascularization and healing [99,100]; in particular, the macrophage switch from the inflammatory to resolving phenotype is an essential step. In fact, in patients with non-healing and diabetic venous ulcers, failure in the M1-to-M2 switch results in local chronic inflammation with impaired healing progression [101]. Interestingly, gene regulation by estrogen is a key mediator of age-related delayed human wound healing [24]. The beneficial effects of estrogen on cutaneous healing are, in part, mediated through macrophage ERα, and estrogen fails to promote alternative macrophage activation in the absence of ERα in vitro [102]. Thus, we propose that estrogen acts as a reprogramming stimulus that accelerates macrophage transition towards a resolving, reparative phenotype [93,103].

Local and systemic metabolism is integrated at the cellular level to regulate immune cell function. By interacting with ER subtypes as discussed in Section 6 below, estrogen also affects metabolic reprogramming in macrophages, which accompanies different activation pathways in response to microenvironmental cues [15,32,81]. It is worth noting that similarities in metabolic reprogramming of macrophages, other immune cells and endothelial cells are emerging [104]. Hence, new insights in immunometabolism can be translated to the clinic to improve current treatments and develop novel therapies for metabolic diseases, inflammation, autoimmunity, and cancer.

These findings point to a complex and partially unresolved role of estrogen in immune and inflammatory responses [98]. Here we suggest that the duality in the action of estrogen on monocyte/macrophages cytokine production depends on many factors including the stimulus triggering the inflammatory response (endogenous or exogenous antigens), the target organ, the different estrogen concentration and ER expression patterns in tissues.

## 6. Estrogen Receptors in the Monocyte/Macrophage System

Recently, ER expression in human monocytes and macrophages has been investigated, increasing the number of pieces of this already complex puzzle. Both cell types express all ERs (Figure 1). Human primary monocytes express the ERα 36-kDa splice variant and GPER1 in a sex-independent manner [43], and these are physically associated. Macrophages have a higher ERα expression and lower ERβ expression than monocytes, and treatment with E2 in monocytes and in human macrophages in vitro induces an increase in ERα expression in macrophages, but not in monocytes [5]. Deficiency of ERα, but not of ERβ, increased TNF-α production by mouse peritoneal macrophages in response to bacterial stimuli, suggesting a prominent role of ERα in mediating the anti-inflammatory effects of estrogen [32,93,96]. Moreover, treatment with the selective GPER1 agonist G-1 is able to inhibit LPS-induced TNF-α production in human macrophages [105]. GPER1 also affects macrophage function via decreasing the expression of TLR4 [106]. In another recent study, it has been demonstrated that E2 confers protection against LPS/NF-κB–induced inflammation, with a role for ERα and GPER1 in mediating these anti-inflammatory properties [43]. In this study, treatment with both ICI 182,780, an ER antagonist/GPER agonist, and G15, a GPER antagonist, blocked the effects of E2. Studies about ERβ and macrophage function are limited; Kramer and colleagues [107] showed that ERβ suppresses CD16 expression with no effect on the activation of MAPKs and NF-κB, while Xing et al. [94] demonstrated an opposite effect showing the ability of selective ERβ activation to inhibit expression of inflammatory mediators. A recent study in human macrophages demonstrated that LPS is able to increase ERα phosphorylation but has no effect on ERβ activation [108]. This study also showed that macrophages isolated from males are more sensitive to the LPS effects than those from females.

As noted above, E2 is able to modulate the activation of different macrophage immune phenotypes [103,109]. The deletion of ERα in hematopoietic cells in mice causes an inability to induce the alternative phenotype in IL-4-stimulated macrophages, and induces high levels of inflammation and insulin resistance, suggesting that ERα is involved in the control of inflammation [110]. Defects in macrophage function due to myeloid-specific ERα deletion also lead to a variety of metabolic disorders including obesity and increased atherosclerosis [110]. Toniolo and colleagues demonstrated that in vitro isolated macrophages stimulated for 48 h with LPS and interferon (IFN)-γ show decreased ERα expression (with unchanged ERβ and GPER-1), and that pre-treatment with E2 counteract the LPS/IFNγ-mediated down-regulation of M2 markers, suggesting that female hormones modulate macrophage immune phenotypes [93]. The observation of a transient up-regulation of ERα mRNA in human macrophages following treatment with IL-4/IL-13 [93] as well as in mouse macrophages treated with IL-4 [103] suggests that this IL-4 effect is well conserved in mammals and may be functionally relevant to the inhibition of the pro-inflammatory response. By using a transcriptomic approach in peritoneal mouse macrophages, Pepe and colleagues recently reported that E2 promotes an anti-inflammatory and pro-resolving macrophage phenotype, which converges on the induction of genes related to macrophage alternative activation and on IL-10 expression in vivo [109]. 

The regulation of the immune response to infection or tissue damage is a complex interplay of multiple factors, but it has long been recognized that estrogen steers the innate and adaptive immune systems at various levels. Thus, we believe that pharmacological targeting of macrophage estrogen pathways may restore the impaired resolution of inflammation associated with aging and chronic inflammatory disease.

## 7. Estrogen in Women’s Health

It has been reported that young women generally have much lower rates of cardiometabolic disease than men. However, midlife women lose this apparent protection during the menopausal transition, so that cardiometabolic disease is most common in post-menopause than any other stage of a woman’s lifespan. In fact, fundamental aspects of metabolic homeostasis are regulated differently in males and females [16,31,111], and influence both the development of disease and the response to pharmacological intervention. Estrogen effects on the cardiovascular system include the modulation of inflammatory response and immune cell function. Aging is characterized by systemic inflammatory changes and organ dysfunction. In females, loss of estrogen makes these changes more intense [112]. Menopause is associated with an increased risk of cardiovascular and metabolic disease largely due to post-menopausal estrogen reduction. For instance, changes in the metabolism of sex hormones lead to accumulation of excess fat in intra-abdominal adipose tissue [15,113]. Post-menopausal women have an abrupt acceleration of atherosclerosis. Although restoration of estrogen would seem to be protective, double-blind clinical studies on the use of estrogen replacement have not shown a benefit in terms of e.g., reduced mortality (reviewed in [114]).

Sex steroid hormones alter the biology of vessel wall cells and the inflammatory cells that accrue as atherosclerosis progresses differently in the early versus later stages of the disease [52]. Hence, the beneficial effects of menopausal hormone therapy in preventing atherosclerotic cardiovascular disease occur only if therapy is initiated before the development of advanced atherosclerosis. Proof of this concept has come from a randomized trial showing that initiation of menopausal hormone therapy in women early after menopause significantly reduces the risk of the combined endpoint of mortality, myocardial infarction or heart failure without resulting in an increased risk of breast cancer or stroke [115]. This suggests that inflammatory pathways should remain an important therapeutic target of estrogen for treating women close to the onset of menopause.

An age relationship of estrogen–monocyte/macrophage number and function has long been identified, which may have several implications for postmenopausal health [112,116]. Studies in human macrophages derived from men and post-menopausal women treated in vitro with E2 highlight that E2 has no influence on the expression of TNF-α, IL-6 and IL-1β, regardless of gender [117,118]. However, the work of Toniolo and colleagues on macrophages derived from women in fertile or menopausal state showed that the response to M2-associated stimuli (IL-4/IL-13) is markedly impaired in macrophages from post- vs. pre-menopausal women, while the response to M1-associated stimuli (LPS/IFNγ) is similar. This results in an increased M1/M2 response ratio in menopausal state, associated with the loss of circulating estrogen [93]. 

The role of E2 in regulating macrophage function is still an evolving topic. In particular, there is interest in understanding how E2 levels in vivo influence the activation of macrophage phenotypes in physiological conditions at different stages of the menstrual cycle as well as in pathological conditions associated with changes in circulating estrogen levels. This piece fits into the broader puzzle of how estrogen pathways impact on macrophage function and, consequently, on immune response, angiogenesis, wound healing and metabolism (Figure 1). Further research on gender differences in the immune response and the onset and progression of autoimmune disease will allow the identification of new preventive strategies and personalized therapeutic approaches for treatment of these immuno-mediated disorders.

## 8. Conclusions

The role of estrogen and its multiple receptors in health and disease is heterogeneous. This makes trying and putting the numerous puzzle pieces together a rather complex task. The protection against cardiovascular disease in women during reproductive age is related, at least in part, to estrogen since endogenous E2 levels and ER expression differ considerably between sexes. Estrogen prevents endothelial dysfunction and atherosclerosis by promoting endothelial healing and increasing angiogenesis. The number of puzzle pieces and with them our knowledge of the mechanisms of estrogen action is growing (Figure 1). Today, it is clear that the combined rapid and genomic effects of estrogen are critical to its overall function; however, these interactions are complex and involve multiple receptor subtypes, both intracellular and membrane-associated. Pharmacological research is poised to design ER ligands that can drive specific transcriptional outcomes, including pathway- and tissue-selective signaling. Targeting specific ERs in the cardiovascular system and fitting together the entire puzzle may result in novel and possibly safer therapeutic options for cardiovascular protection.

## Abbreviations

AF	activator factor
cAMP	cyclic adenosine monophosphate
E2	17β-estradiol
eNOS	endothelial nitric oxide synthase
ERE	estrogen response element
ER	estrogen receptor
FAK	focal adhesion kinase
G-1	(±)-1-[(3aR*,4S*,9bS*)-4-(6-Bromo-1,3-benzodioxol-5-yl)-3a,4,5,9b-tetrahydro-3H-cyclopenta[c]quinolin-8-yl]-ethanone
GPER1	G-protein-coupled ER
HUVEC	human umbilical vein endothelial cells
IL	interleukin
IFNγ	interferon-γ
LBD	Ligand-Binding Domain
LPS	lipopolysaccharide
MAPK	mitogen-activated protein kinase
MCP-1	monocyte chemoattractant protein-1
NF-κB	nuclear factor kappa-light-chain-enhancer of activated B cells
NO	nitric oxide
PFKFB3	phosphofructokinase-2/fructose-2,6-bisphosphatase-3
PI3K	phosphatidylinositol-3-kinases
TNF-α	tumor necrosis factor α
VEGF	vascular endothelial growth factor
